# Fluid Secretion by Malpighian Tubules of *Rhodnius prolixus*: Neuroendocrine Control With New Insights From a Transcriptome Analysis

**DOI:** 10.3389/fendo.2021.722487

**Published:** 2021-08-26

**Authors:** Ian Orchard, Jimena Leyria, Areej Al-Dailami, Angela B. Lange

**Affiliations:** Department of Biology, University of Toronto Mississauga, Mississauga, ON, Canada

**Keywords:** insect, GPCR, neurohormones, second messengers, epithelial transport

## Abstract

*Rhodnius prolixus* (the kissing bug and a major vector of Chagas disease) is an obligate blood feeder that in the case of the fifth instar consumes up to 10 times its unfed body weight in a single 20-minute feed. A post-prandial diuresis is initiated, within minutes of the start of gorging, in order to lower the mass and concentrate the nutrients of the meal. Thus, *R. prolixus* rapidly excretes a fluid that is high in NaCl content and hypo-osmotic to the hemolymph, thereby eliminating 50% of the volume of the blood meal within 3 hours of gorging. In *R. prolixus*, as with other insects, the Malpighian tubules play a critical role in diuresis. Malpighian tubules are not innervated, and their fine control comes under the influence of the neuroendocrine system that releases amines and neuropeptides as diuretic or antidiuretic hormones. These hormones act upon the Malpighian tubules *via* a variety of G protein-coupled receptors linked to second messenger systems that influence ion transporters and aquaporins; thereby regulating fluid secretion. Much has been discovered about the control of diuresis in *R. prolixus*, and other model insects, using classical endocrinological studies. The post-genomic era, however, has brought new insights, identifying novel diuretic and antidiuretic hormone-signaling pathways whilst also validating many of the classical discoveries. This paper will focus on recent discoveries into the neuroendocrine control of the rapid post-prandial diuresis in *R. prolixus*, in order to emphasize new insights from a transcriptome analysis of Malpighian tubules taken from unfed and fed bugs.

## Introduction

Insects have overcome the problems associated with a large surface area to volume ratio by evolving a variety of adaptations to maintain an appropriate state of hydration. Thus, insects balance the water lost during evaporation, respiration, and excretion, with water gained during feeding and metabolism, or in extreme cases, from the atmosphere (1, 2). Water loss in particular is tightly controlled and regulated, since terrestrial insects typically need to conserve water. However, the availability of water does vary greatly throughout the life of an insect and there are times when insects need to eliminate fluid rather than conserve it; for example, following feeding (especially in hematophagous insects consuming large blood meals), during eclosion/ecdysis when hemolymph volume is increased for wing expansion, and during flight to eliminate metabolically-generated water (1, 3).

The excretory system in insects is naturally involved in the elimination of excess fluid and waste, and typically includes the Malpighian tubules and hindgut. However, in some blood feeding insects, before the Malpighian tubules can excrete parts of the blood meal, the midgut epithelium must absorb the fluid to be excreted, and so in these cases the midgut is considered part of the excretory system. Excretory water loss and the salt composition of the fluid (incorporating diuresis) is ultimately determined by the rate at which any fluid is absorbed into the hemolymph from the midgut, the rate at which fluid is then secreted by the Malpighian tubules from the hemolymph into the lumen, the rate at which Malpighian tubules may reabsorb ions from lumen to hemolymph prior to delivery into the hindgut, the rate at which digestive fluid enters the hindgut from the midgut, and then the rate of reabsorption of fluid across the hindgut, prior to excretion. The balance between these processes determines final volume and composition of the urine and will vary between insect species.

The Malpighian tubules represent one of the most important tissues involved in diuresis and can transport fluid at very high rates. It is worth noting that unlike the hydrostatic filtration used by the vertebrate nephron, the secretion process in the Malpighian tubules is driven by an apical V-ATPase that establishes an electrochemical gradient, coupled with active transport of Na^+^ and K^+^ with water following passively through aquaporins (4–6).

It is now well understood that diuresis is under either neuroendocrine or direct neural control; the fine control allowing for the maintenance of hemolymph volume, osmotic, and ionic composition, whilst eliminating excess water and ions, nitrogenous waste, and toxic substances from the insect (1). With respect to neuroendocrine control, diuretic and antidiuretic hormones commonly act in concert on the Malpighian tubules and hindgut *via* G protein-coupled receptors (GPCRs), with diuretic hormones acting primarily on Malpighian tubules to increase urine secretion and antidiuretic hormones acting primarily on the hindgut to stimulate fluid reabsorption (1). In the case of *Rhodnius prolixus*, the diuretic hormones that act upon the Malpighian tubules to stimulate fluid secretion (7), simultaneously also act on the midgut to stimulate absorption of fluid from the blood meal into the hemolymph. In addition, the antidiuretic hormone in *R. prolixu*s acts upon the anterior midgut and Malpighian tubules to reduce urine production (8).

The post-genomic era has brought forth great advances in our understanding of the diuretic processes in insects, enabling the identification and quantification of thousands of target genes in a single experiment, as well as enabling comparative genomic studies. Microarray and transcriptome analyses of Malpighian tubules have also reinforced the major roles that Malpighian tubules play in detoxification and immunity, and in tolerance to stress and desiccation (2, 4, 9–13). The *R. prolixus* genome has been sequenced and reported (14), bringing the post-genomic era to bear on this historic model insect.

Much has been discovered about Malpighian tubules using these advanced molecular technologies, including identification of diuretic/antidiuretic hormones, their potential GPCRs, the second messenger systems utilized, mode of action at the molecular and cellular level, and potential biological relevance. The goal of the present study is to conduct a transcriptome analysis of Malpighian tubules taken from unfed and fed kissing bugs, *R. prolixus*. The remainder of this Introduction provides a comprehensive review of short-term physiological events, with an emphasis on GPCRs associated with diuresis, in order to better understand the new insights gained from the transcriptome analysis.

### Rhodnius prolixus

*Rhodnius prolixus*, a.k.a. the kissing bug, for its penchant of taking a blood meal from the faces of humans, has played a vital and pivotal role in insect comparative physiology and endocrinology for almost 100 years (15, 16). Davey (17), a student of Wigglesworth and who himself has had a profound influence on the discipline of insect endocrinology, emphasizes that “it is one of the model insects on which the foundations of insect physiology were built”. The kissing bug is also medically important, since it is a principal vector of *Trypanosoma cruzi*, the causative agent of Chagas disease, a neglected disease endemic to Central and South America. *Rhodnius prolixus* may ingest human blood infected with *T. cruzi*, with the parasite eventually residing in the insect’s hindgut. From here, the parasite is passed on to the next human in the urine/feces that are eliminated after gorging. Hence the control of diuresis is an important link in the transmission of Chagas disease.

**Figure 1:**
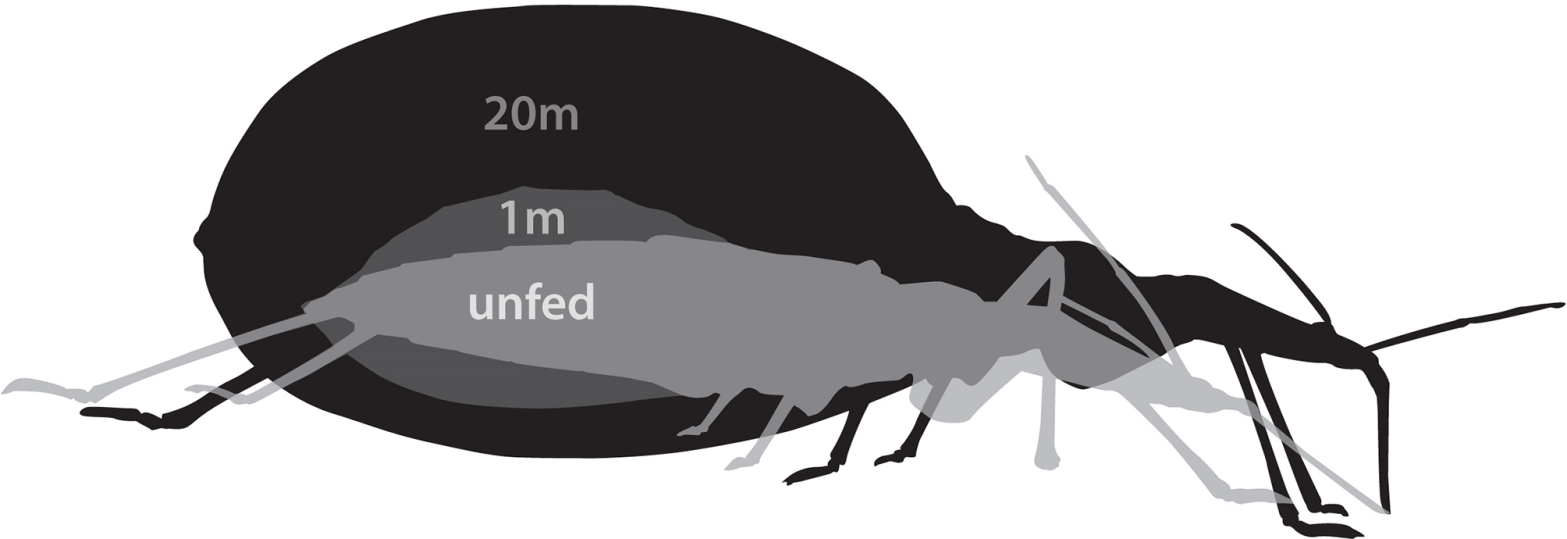
Drawing depicts the change in body shape of fifth instar *Rhodnius prolixus* during blood gorging. Note that as the insect transitions from an unfed, through 1 minute (1 m) and 20 minutes (20 m) of blood gorging, body shape expands in all directions, including anterior to posterior, to accommodate a blood meal that can be up to 10 times the insect’s initial body mass.

**Figure 2:**
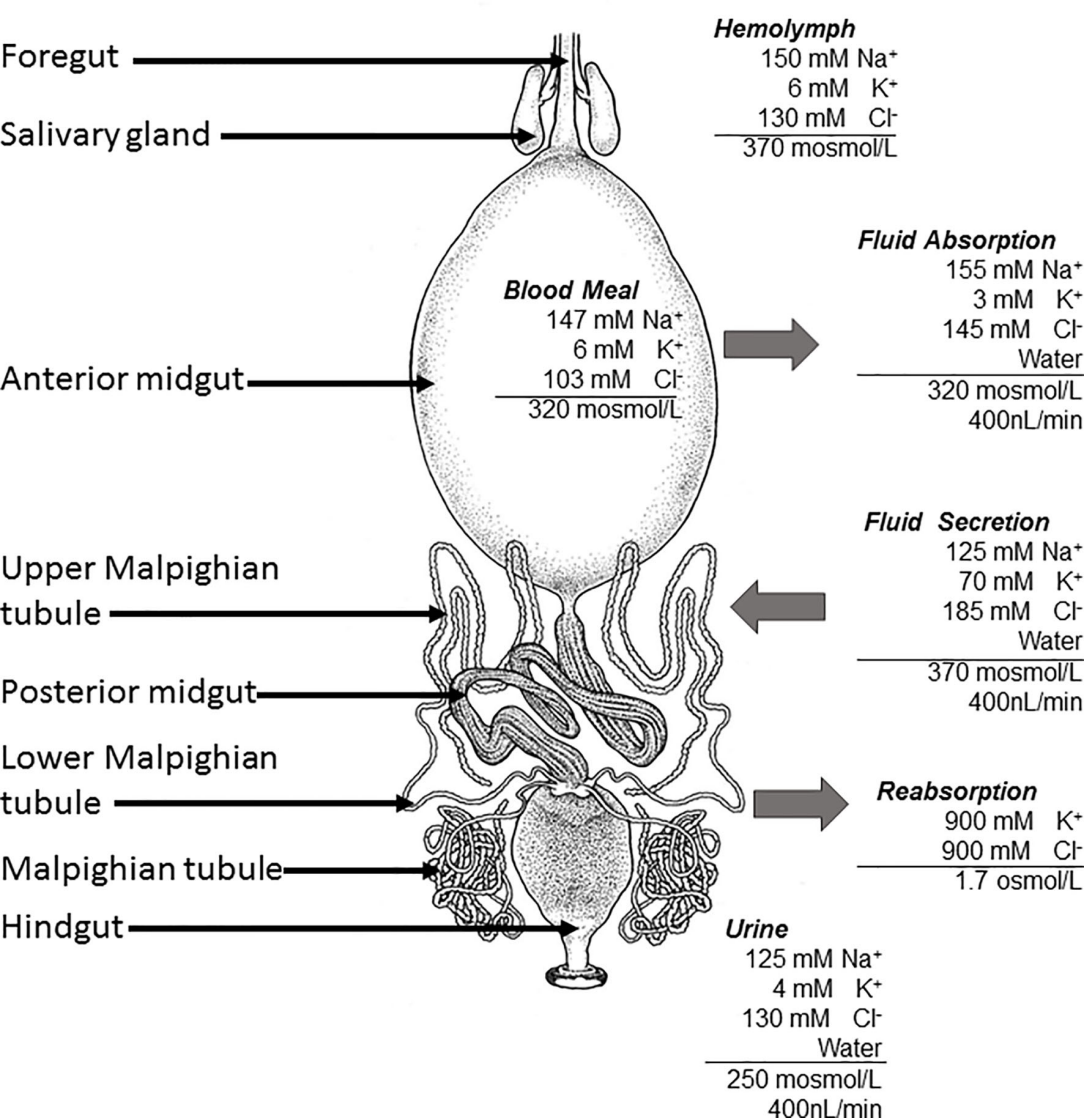
The digestive system of *Rhodnius prolixus*, including the tissues involved in diuresis. The ionic composition of the blood meal and hemolymph are shown, and arrows indicate movement of water and ions during post-prandial diuresis. Primary urine is eliminated *via* the hindgut (data taken from 18).

The five instars and adults of both sexes of *R. proli*xus are obligate blood feeders and require a single blood meal to trigger endocrinological events associated with development, growth, and reproduction. In the case of the penultimate instar, gorging takes between 15-20 minutes and can increase the insect’s body weight up to 8-10 fold. To accommodate this blood meal within the anterior midgut, the cuticle undergoes plasticization, and the insect is stretched in all planes, like a balloon (**Figure 1**). These insects are more vulnerable to predation and exposure to potential toxic blood components, and so they must rapidly eliminate excess water and salts to reduce their own volume and eliminate waste products. Thus, the considerable stress imposed by this blood gorging is alleviated by rapid physiological and endocrinological changes that lower the mass of the blood meal and concentrate the nutrients in the anterior midgut, whilst preserving the volume, ionic, and osmotic balance of the hemolymph see (7, 18, 19)]. This process begins within minutes of the start of gorging and involves the transport of NaCl and water (isosmotic with the blood meal) across the anterior midgut epithelium into the hemolymph (**Figure 2**). At the same time, the 4 Malpighian tubules become involved. The distal (secretory) portion of the Malpighian tubule secretes a fluid that is isosmotic with the hemolymph but containing high NaCl and KCl content. The most proximal (reabsorbing) portion of the Malpighian tubules modifies the fluid by reabsorbing KCl with very little water into the hemolymph. The primary urine entering the hindgut is similar in ionic and osmotic composition to the plasma of the blood meal, and this urine is voided without any compositional modifications by contractions of the hindgut (**Figure 2**). Thus, *R. prolixus* rapidly excretes a fluid of high NaCl content that is hypo-osmotic to the hemolymph (for details see 7, 19, 20). Diuresis occurs at such a very rapid rate, that within 3 h of gorging, the insect can eliminate up to 50% of the volume of the blood meal; equivalent to 10 times the insect’s hemolymph volume. Thus, *R. prolixus* Malpighian tubules represent an excellent model for studying the control of fluid and ion secretion in view of the impressive fluid movement achieved in such a rapid way. As with other insect Malpighian tubules, *R. prolixus* tubules are also useful models for studying epithelial transport and much is known about the various transporters and co-transporters involved in diuresis (for reviews see 1, 3–5). Importantly, insect Malpighian tubules are not innervated, but are under neurohormonal control, and in three cases, possibly from hormones produced by the tubules themselves (21–23). They therefore also provide models for studying hormone-signaling pathways regulating epithelial transport.

### Diuretic and Antidiuretic Hormones and Their GPCRs in *R. prolixus* – A Current Model

In the pre-genomic era, diuretic and antidiuretic factors were discovered using high performance liquid chromatography and sequencing *via* Edman degradation, later matrix assisted laser desorption ionization-time of flight mass spectrometry, coupled to sensitive biological assays (for discussion see 1, 3). Bioassays were established (24) whereby factors could be tested for their ability to increase or decrease fluid secretion *in vivo*, or Malpighian tubule secretion *in vitro*, or elevate second messengers [often cyclic AMP (cAMP)] *in vitro*. Alternative assays have also been developed, including electrophysiology and monitoring the influence of these factors on ionic currents or transepithelial potentials (25). The post-genomic era has enabled a complementary approach of reverse engineering; that is, reverse genetic and endocrinological approaches enabling identification of candidate genes/transcripts and subsequently deriving their biological function and relevance. While the reverse approach is proving successful, especially for the GPCRs and their ligands (26), bioassays are still vital tools to be used alongside these modern technologies.

Diuretic and antidiuretic hormones in insects are mainly neuropeptides belonging to several peptide families, although the biogenic amines, serotonin, and tyramine, have been shown to be diuretic hormones in *R. prolixus* and *Drosophila melanogaster* respectively. Diuretic hormones act directly on the Malpighian tubules, whereas antidiuretic hormones can act on midgut, Malpighian tubules, or hindgut, depending upon the species (3, 7). A relatively large number of peptide families have been shown to possess diuretic or antidiuretic activity in a wide range of insect species, and further support for their true biological relevance comes from the presence of their cognate GPCRs in tissues associated with these processes. These studies also reinforce the notion that these hormones have multiple functions in each insect and appear to control quite disparate activities associated with a common behavior, such as feeding, and with stress tolerance [see (7)].

**Figure 3:**
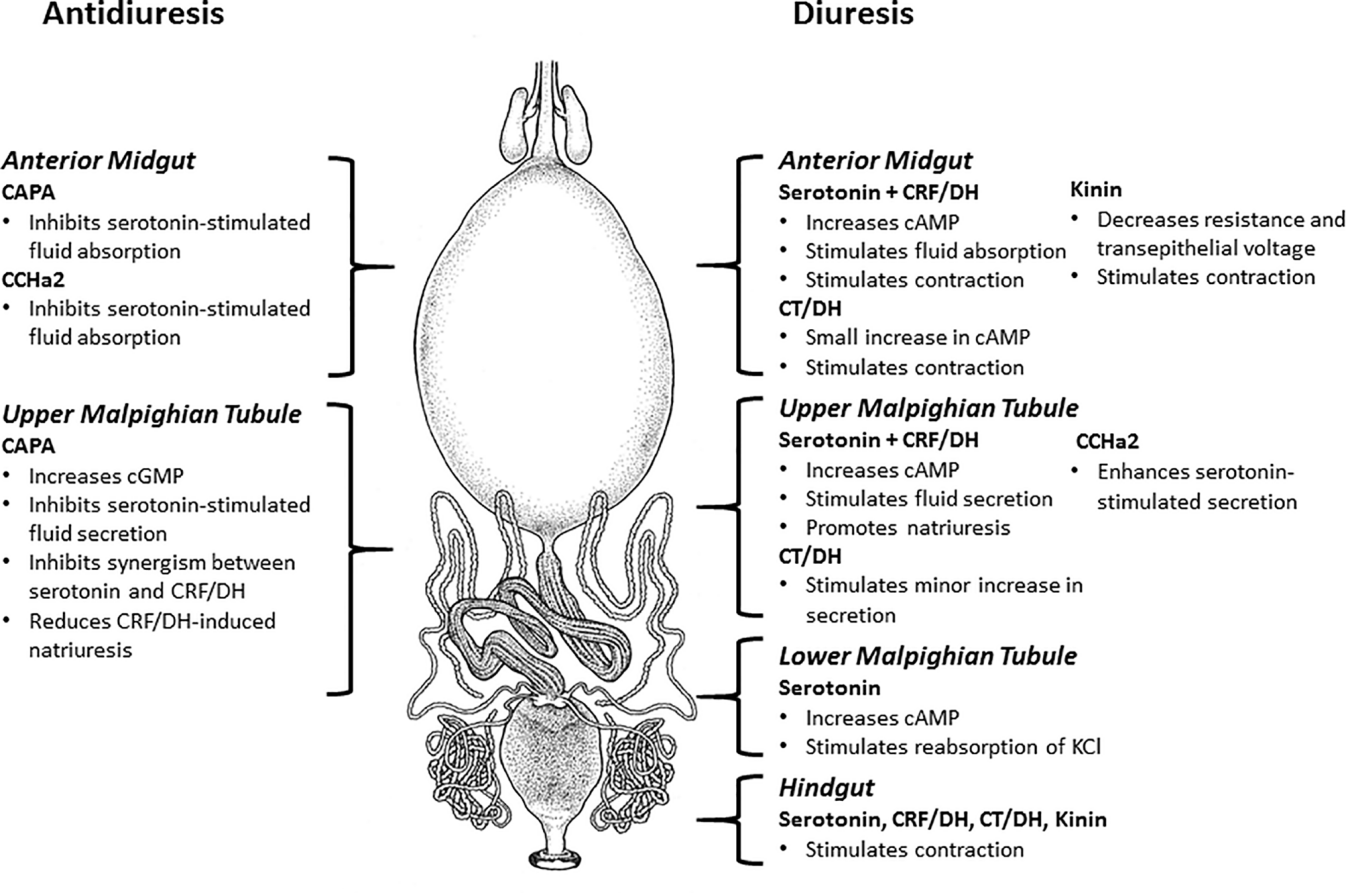
Model depicting the neuroendocrine control of post-prandial diuresis and its termination by antidiuretic hormones in *Rhodnius prolixus*. Diuresis is controlled by serotonin and Rhopr-CRF/DH (corticotropin-releasing factor-related diuretic hormone) acting upon the anterior midgut (to stimulate absorption), and simultaneously on the distal (upper) portion of the Malpighian tubules (to stimulate secretion). Serotonin alone acts upon the proximal (lower) portion of Malpighian tubules to stimulate reabsorption of KCl. Rhopr-CT/DH (calcitonin-related diuretic hormone), which is co-released from the same neurosecretory cells that release serotonin, stimulates only a small increase in secretion by Malpighian tubules. Rhopr-K (Kinin) which is co-released from the same neurosecretory cells that release Rhop-CRF/DH decreases resistance and transepithelial voltage of the anterior midgut but does not stimulate absorption, and also does not stimulate secretion by Malpighian tubules. CCHa2 enhances serotonin-stimulated secretion by Malpighian tubules. Serotonin, Rhopr-CRF/DH, Rhopr-CT/DH and Rhopr-K all stimulate contractions of anterior midgut and contractions of hindgut. Diuresis is terminated by the release of Rhopr-CAPA2 (CAPA) which inhibits serotonin-stimulated absorption by anterior midgut, serotonin stimulated secretion by Malpighian tubules, and also eliminates the synergism between serotonin and Rhopr-CRF/DH. CCHa2 inhibits serotonin-stimulated absorption across the anterior midgut. References in text.

A model for the actions of diuretic/antidiuretic hormones in *R. prolixus* is shown in **Figure 3** and is discussed below.

#### Diuretic Hormones

##### Serotonin (5-Hydroxtryptamine, 5HT) and Its Receptor

Serotonin was shown to be a potent diuretic factor in *R. prolixus*, mimicking the effects of a putative diuretic neuropeptide (27). Later, serotonin was shown to be a true diuretic hormone (28, 29), which is released into the hemolymph from the abdominal neurohemal sites of 5 dorsal unpaired median (DUM) neurons in the mesothoracic ganglionic mass (MTGM) of the central nervous system (CNS). Serotonin stimulates absorption of fluid across the anterior midgut, and secretion by distal Malpighian tubules *via* a cAMP-dependent mechanism, and stimulates proximal tubules to reabsorb KCl (7, 29).

The titer of serotonin in the hemolymph of *R. prolixus* increases from a level of about 7 nM in unfed insects to a peak of 115 nM 5 minutes after the onset of gorging (28). The titer drops to 40 nM by the time gorging is completed, 10 - 15 minutes later, drops again to 22 nM by 20 min, and then remains elevated at 15 nM for at least 24 h. The peak titer of serotonin in the hemolymph is sufficient to activate the *R. prolixus* serotonin GPCR (Rhopr5HTR2b), induce cAMP elevation, and stimulate maximum fluid transport in Malpighian tubules and anterior midgut *in vitro* (7, 29, 30). In addition, serotonin stimulates contractions of the hindgut, thereby aiding in the voiding of the urine/feces (29).

Regarding the anterior midgut, Farmer et al. (31) used *in vitro* preparations of this tissue to demonstrate transport of a NaCl-based fluid which is isosmotic to the blood meal. Serotonin elevates cAMP and stimulates absorption of fluid across the anterior midgut, transporting a NaCl rich fluid from the lumen (containing the blood meal) into the hemolymph. Na^+^ and Cl^-^ move passively from the lumen into the epithelial cells, with active transport of Na^+^ into the hemolymph using a Na^+^/K^+^-ATPase. Water and Cl^-^ are believed to move passively (31, 32). The peak of hemolymph titer of serotonin is capable of increasing absorption at the anterior midgut by about 4-fold.

For Malpighian tubules, electrophysiological measurements indicate that serotonin, and its second messenger cAMP, can maximally stimulate fluid secretion by driving the active transport of K^+^ and/or Na^+^ into the distal tubule lumen accompanied by Cl^-^, with water following osmotically (33, 34). A V-ATPase generates a proton gradient in the apical membrane that drives K^+^/Na^+^ transport into the lumen through alkali cation/proton antiporters. K^+^/Na^+^ are taken up across the basolateral membrane *via* ion channels and by cation/Cl^-^ co-transporters and/or K^+^/Na^+^-ATPase active transport.

The most proximal portion of the Malpighian tubules (the lowermost 30% of the proximal tubule) in *R. prolixus* is stimulated by serotonin and this portion reabsorbs KCl (but not water), and it is suggested that K^+^ is pumped from the lumen into the epithelial cells by an H^+^/K^+^-ATPase, with K^+^ leaking from the cells into the hemolymph *via* Ba^2+^-sensitive K^+^ channels. Cl^-^moves from lumen to epithelial cells through a $\text{C}{\text{l}}^{-}{\text{/HCO}}_{3}^{-}$ exchanger, then exits into the hemolymph through basolateral Cl^-^ channels (3, 35, 36).

**Figure 4:**
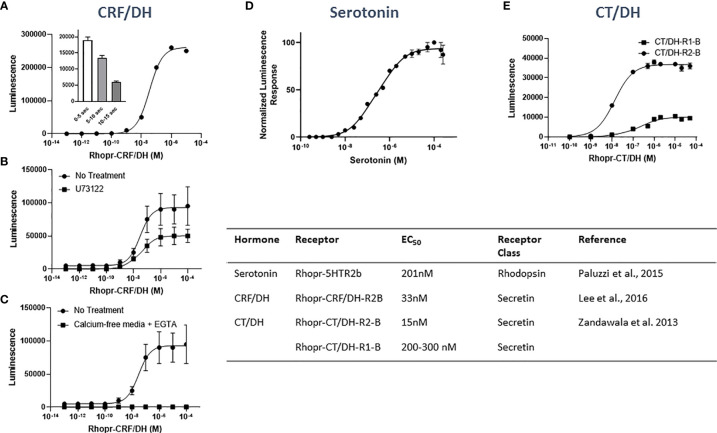
Heterologous functional assay and properties (Table insert) of GPCRs for the diuretic hormones associated with Malpighian tubules of *Rhodnus prolixus*. **(A)** Rhopr-CRF/DH-R2B transiently expressed in CHO/G16 cells is dose-dependently activated by Rhopr-CRF/DH. Inset illustrates the kinetics of response to 10^− 5^ M Rhopr-CRF/DH in 5 s intervals over a period of 15 s. Note the fast response. **(B)** Functional assay of Rhopr-CRF/DH-R2B transiently expressed in HEK293/CNG cells. Dose-response curves showing the effect of Rhopr-CRF/DH on Rhopr-CRF/DH-R2B activation in the presence or absence of 10 μM U73122. The response is still evident in the presence of U73122 illustrating that the PLC/IP_3_ pathway is not the natural second messenger pathway. **(C)** Dose-response curves showing the effect of Rhopr-CRF/DH on Rhopr-CRF/DH-R2B activation in the presence or absence of extracellular calcium. Note the response is completely eliminated in the absence of extracellular calcium, illustrating that Rhopr-CRF/DH is naturally elevating cAMP, which in this modified cell line opens up the cyclic nucleotide-gated (CNG) channel allowing extracellular calcium to enter the cell and be observed through luminescence. **(D)** Heterologous functional assay of the *R. prolixus* serotonin type 2b receptor (Rhopr5HTR2b) in CHOK1-aeq cells. Dose-response curve demonstrating activity of serotonin on the expressed Rhopr5HTR2b receptor. **(E)** Heterologous functional assay of *R. prolixus* CT/DH receptor isoforms (Rhopr-CT/DH-R1-B and Rhopr-CT/DH-R2-B transiently expressed in HEK293/CNG cell lines. Dose-dependent effect on the bioluminescence response after addition of Rhopr-CT/DH to HEK293/CNG cells illustrates a much greater response from Rhopr-CT/DH-R1-B. Graphs redrawn from references (30, 38, 39) in the Table insert.

Although 5 serotonin receptors have been described in insects (37), so far only one *R. prolixus* serotonin GPCR has been cloned and characterized (Rhopr5HTR2b). Rhopr5HTR2b transcript is most highly expressed in Malpighian tubules, but also highly expressed in foregut, hindgut, salivary glands, and CNS, although interestingly is present, but not highly abundant, in anterior midgut (30). This receptor is a rhodopsin-like family A member and was verified as a *bona fide* target of serotonergic signaling using a heterologous cell assay based upon recombinant Chinese Hamster Ovary (CHO)K1-aeq cells stably expressing the jellyfish photoprotein apoaequorin (**Figure 4**). CHOK1-aeq cells transiently expressing Rhopr5HTR2b yielded dose-dependent luminescence responses to serotonin with threshold activity in the low nanomolar range. The vertebrate serotonin receptor type-2 agonist, alpha-methyl serotonin, yielded a similar dose-dependent luminescence response, albeit with lower potency.

##### Corticotropin-Releasing Factor-Related Diuretic Hormone and Its Receptor

Evidence for a neuropeptidergic diuretic hormone in insects was first presented by Maddrell (40) in *R. prolixus*. Such a neuropeptide was finally isolated and sequenced in *Manduca sexta* (41) and found to have sequence similarity to the vertebrate corticotropin-releasing factor (hormone), and so this family in insects is referred to as the corticotropin-releasing factor-related diuretic hormone (CRF/DH). Corticotropin-releasing factor, urotensin-1, urocortin, and sauvagine form a family of neuropeptides now known to be present in nematodes, arthropods, mollusks, tunicates, and the chordates (42). The list of homologous peptides in a wide range of insect species has now grown (*de novo* sequenced or predicted through bioinformatics/molecular techniques). Interestingly, the insect CRF/DH family is quite structurally diverse whereas the vertebrate CRF family has high sequence conservation (1, 3).

Rhopr-CRF/DH in *R. prolixus* is a true diuretic hormone, released into the hemolymph from abdominal neurohemal sites of lateral neurosecretory cells in the MTGM at gorging with titers reaching 5 nM at 1 h following gorging (38). Rhopr-CRF/DH elevates cAMP, induces similar electrophysiological changes as serotonin, and stimulates maximum fluid secretion from *R. prolixus* distal Malpighian tubules with an EC_50_ of 3 nM (43–45). However, Rhopr-CRF/DH does not stimulate KCl reabsorption from the proximal Malpighian tubules (46), although any modulating effects of Rhopr-CRF/DH on serotonin’s action have not been examined.

Rhopr-CRF/DH significantly increases cAMP content, and the rate of absorption across the anterior midgut, indicating that like the distal Malpighian tubules, the anterior midgut is under the control of both serotonin and Rhopr-CRF/DH, each acting *via* cAMP (43, 47). In addition, as with serotonin, Rhopr-CRF/DH also increases the frequency of contractions of the hindgut and so may be involved in voiding of the feces/urine (19).

The sequencing of the first CRF/DH in insects from *M. sexta* was followed by cloning the *M. sexta* CRF/DH receptor (48), and subsequently the receptors in *Acheta domesticus* (49), *D. melanogaster* (50) and later many other insect species, including *R. prolixus* (38). The Rhopr-CRF/DH receptor (Rhopr-CRF/DH-R2B) transcript is expressed more highly in the distal Malpighian tubules than in the proximal Malpighian tubules of *R. prolixus* and is also expressed in the anterior midgut, hindgut, and many other tissues (38). These GPCRs belong to the secretin family B1 that primarily uses cAMP as their second messenger. This link to cAMP was confirmed in *R. prolixus* (**Figure 4**) for one of the two Rhopr-CRF/DH receptors (Rhopr-CRF/DH-R2B) using a heterologous cell system (38). Human embryonic kidney (HEK)-293 cells stably expressing a modified cyclic-nucleotide-gated (CNG) channel (HEK293/CNG) were used to test if Rhopr-CRF/DH-R2B naturally works through the phospholipase C/inositol trisphosphate (PLC/IP_3_) pathway or through the adenylate cyclase (cAMP) pathway. The results indicate that cAMP is the second messenger. This receptor has also been transiently expressed in CHO/G16 cells and ligand-receptor interaction monitored using a Ca^2+^ mobilization assay. EC_50_ values for Rhopr-CRF/DH in this cell assay were approximately 36nM (**Figure 4**).

##### Synergism Between Serotonin and Rhopr-CRF/DH on Malpighian Tubule Secretion

Even though fluid crosses the anterior midgut of *R. prolixus* into the hemolymph rapidly, at about 400 nl/min, there are only small changes in the composition of the hemolymph. This is due to the distal Malpighian tubules secreting fluid at an equal rate from the hemolymph into the lumen (20). The distal Malpighian tubules secrete a fluid containing 2:1 Na^+^:K^+^ with ion transport stimulated 1000-fold after a blood meal is imbibed. The epithelial cells of the Malpighian tubules are stimulated within minutes of the onset of gorging by the diuretic hormones released from the abdominal nerve neurohemal sites of the MTGM (51). This stimulation and diuresis persist for about 3 h. The hemolymph titers of these two diuretic hormones after gorging are certainly high enough to act upon distal Malpighian tubules to stimulate secretion.

But why does *R. prolixus* use two diuretic hormones for rapid post-prandial diuresis? One answer lies in the synergism between the two neurohormones. Synergism is defined as an interaction or cooperation of two hormones resulting in a combined effect greater than the sum of their individual effects, a definition that matches the need for rapid post-prandial diuresis. Synergism steepens the dose-response curve and shifts it towards lower concentrations, providing a functionality for having multiple diuretic hormones. The result is that the Malpighian tubules can be stimulated more quickly and at lower concentrations of each of the diuretic hormones. Rhopr-CRF/DH, serotonin and dibutyryl cAMP are each capable of eliciting maximum secretion rates in Malpighian tubules *in vitro* but low doses of Rhopr-CRF/DH and serotonin applied together act synergistically to increase secretion rates *via* the cAMP-signaling pathway (7, 52–54). There is much evidence for Rhopr-CRF/DH and serotonin both working through cAMP, and synergism of adenylate cyclase activity is also seen when a tissue homogenate containing the peptide and serotonin are applied together (54). Synergistic activation of adenylate cyclase has been shown to occur from signals that are received coincidentally from GPCRs and *via* pathways that increase intracellular calcium concentration [see (38)]. With that in mind, Gioino et al. (55) found that intracellular calcium waves were triggered in Malpighian tubules treated with serotonin (and with 8-bromoadenosine 3’,5’-cyclic adenosine monophosphate, 8-Br-cAMP) and these waves were blocked using a cell permeable calcium chelator, 1,2-bis(o-aminophenoxy)ethane-N,N,N′,N′-tetraacetic acid (BAPTA-AM), in calcium-free saline. Serotonin, *via* its receptor, was not directly releasing intracellular Ca^2+^ but serotonin-induced increases in cAMP trigger Ca^2+^ waves mediated through a protein kinase A (PKA) signaling pathway. Hence, 8-Br-cAMP also triggers Ca^2+^ waves and maximum secretion. When the Ca^2+^ waves are blocked, fluid secretion is reduced by 75% with the fluid containing more K^+^ and reduced Na^+^. Interestingly the Ca^2+^ waves are suggested to be initiated in a small number of principal cells, referred to as pioneer cells, and these waves are then propagated to neighboring cells. This suggests that principal cells are not homogeneous, and it is of course possible that serotonin and Rhopr-CRF/DH act upon different cells in the Malpighian tubules. Rhopr-CRF/DH is yet to be tested in these Ca^2+^ assays, although Paluzzi et al. (45) found that both serotonin and Rhopr-CRF/DH activate pathways that are dependent upon intracellular Ca^2+^ and cAMP.

One model for this synergism is the synergistic stimulation of adenylate cyclase activity induced by serotonin and Rhopr-CRF/DH (54). Subsequent calcium waves may contribute to this synergism. The elevated levels of cAMP may activate the PKA-signaling pathway to trigger an increase in IP_3_ that in turn increases the amplitude and/or frequency of calcium waves released intracellularly from storage sites within the cell (55). The elevated calcium levels could then modulate the various transporters to drive increases in diuresis that are larger than the sum of activity produced by low concentrations of the diuretic hormones working independently.

Serotonin is released rapidly into the hemolymph immediately after the onset of gorging and initiates diuresis (28). The titer peaks at 5 minutes and thereafter declines to levels which are below those that stimulate fluid absorption from the anterior midgut or secretion by the distal Malpighian tubules but remain high enough to stimulate reabsorption of KCl across the proximal Malpighian tubules (20 nM serotonin stimulates approximately 70% maximal KCl reabsorption). Release of Rhopr-CRF/DH into the hemolymph occurs later than the release of serotonin and is needed to continue secretion from the distal Malpighian tubules. With haemolymph titers of Rhopr-CRF/DH in the nM range, these would be enough to synergize with the low titers of serotonin present over the 3 h period of rapid post-prandial diuresis.

##### Calcitonin-Related Diuretic Hormone and Its Receptor

The insect calcitonin-related diuretic hormone family (CT/DH) is related to the mammalian calcitonin and calcitonin gene-related peptide hormone system. This family of CT/DHs stimulate, at nM concentrations, Malpighian tubule secretion in a variety of insects and increases cAMP content in the principal cells of *D. melanogaster* tubules. Rhopr-CT/DH, which interestingly is co-localized with serotonin in the 5 DUM cells of the MTGM, increases the rate of secretion to only 1.5% of the maximum rate induced by serotonin or by Rhopr-CRF/DH. For *R. prolixus*, during rapid post-prandial diuresis, this may be considered a small increase relative to serotonin, but it is still a 17-fold increase over the non-stimulated rate, which is naturally, very low (56). The biological significance of this small increase is not known, since in addition, Rhopr-CT/DH does not significantly increase water absorption or short-circuit current across the anterior midgut (47). Any modulatory effects of Rhopr-CT/DH on the other diuretic/antidiuretic hormones have not been investigated.

CT/DH receptors are members of the secretin family B1 GPCRs. *R. prolixus* has two receptor paralogues (Rhopr-CT/DH-R1 and R2). Rhopr-CT/DH-R1 is orthologous to the *D. melanogaster* CT/DH receptor (CG17415) while Rhopr-CT/DH-R2 is orthologous to the *D. melanogaster* receptor (CG4395), an orphan receptor whose ligand had previously been unknown (39). Splice variants are also present, producing Rhopr-CT/DH-R1 (A, B and C) and Rhopr-CT/DH-R2 (A and B). Rhopr-CT/DH-R2B is more sensitive to Rhopr-CT/DH (EC_50_ = 15 nM) than is Rhopr-CT/DH-R1B/C and also yields a much greater amplitude response in a heterologous cell assay [**Figure 4**; (39)]. The “A” splice variants are truncated and non-functional. Interestingly, and in contrast to the study on the *D. melanogaster* receptor, accessory proteins (RAMPs or RCP) are not required for effective signaling. The Rhopr-CT/DH-R2B transcript is expressed in a variety of tissues including Malpighian tubules, but Rhopr-CT/DH-R1 is not (39). In addition, Rhopr-CT/DH-R2 is not expressed in the anterior midgut, explaining the absence of effect of Rhopr-CT/DH on fluid absorption.

##### Rhopr-Kinin and Its Receptor

Insect kinins are found throughout Arthropoda and possess a conserved C-terminal pentapeptide motif of FX_1_X_2_WGamide [see (57)]. A single transcript encoding multiple isoforms has been characterized in many insect species including 12 in *R. prolixus* (58). Kinins are potent diuretic factors in a variety of species (3), but kinins, which are co-localized with the Rhopr-CRF/DH in the posterior lateral neurosecretory cells of the MTGM in *R. prolixus*, have no effect on secretion by Malpighian tubules in *R. prolixus* (59). They do however act as potent stimulators of contraction of hindgut and anterior midgut, and significantly decrease the resistance and transepithelial voltage of the anterior midgut epithelium (47). Thus, kinins may participate in some aspects of diuresis.

The Rhopr-kinin receptor (Rhopr-KR) has been cloned and belongs to the rhodopsin-like family A group of GPCRs (60). High expression of the Rhopr-KR transcript is observed in the CNS and hindgut, with lower transcript expression levels in the remainder of the digestive system, including the anterior midgut, and low expression observed in the Malpighian tubules, explaining the physiological results.

#### Antidiuretic Hormones and the Termination of Diuresis

##### CAPA and Its Receptor

The *M. sexta* cardoacceleratory peptide 2b, Manse-CAP_2b_ (61), was shown to stimulate fluid secretion from Malpighian tubules of *D. melanogaster* (62). The gene/transcript has been discovered in a number of insect species, and in *D. melanogaster* the gene was named *capability* (CG15520) since it was “capable” of encoding CAP_2b_. Typically, three mature peptides are derived from the CAPA transcript. The first two (CAPA1 and 2) normally contain the CAP_2b_ consensus C-terminal sequence of PRVamide, while the third (CAPA3) does not. CAPA1 and 2 are strong diuretic factors in dipterans [see (3)] and have been extensively studied in *D. melanogaster* and shown to act through a Ca^2+^-nitric oxide (NO)-cGMP signaling pathway. This pathway increases V-ATPase activity in the principal cells. However, much lower doses of CAPA have been shown to be antidiuretic in *D. melanogaster* and in *Aedes aegypti* indicating that CAPAs can have dose-dependent opposing effects on diuresis in some dipterans (63–66). CAPAs are also antidiuretic in some other insects, including *R. prolixus* (3, 67).

Diuresis rapidly declines at about 3 h after gorging in *R. prolixus* when enough hypo-osmotic fluid has been excreted. This termination of diuresis avoids excessive loss of water and salts, thereby maintaining volume, ionic and osmotic composition of the hemolymph. Rhopr-CAPA-2 is an antidiuretic hormone allowing finer control over diuresis (67). The diuretic hormones in *R. prolixus* act *via* cAMP, but cGMP content of Malpighian tubules is elevated *in vivo* at a time when diuresis is ceasing. Rhopr-CAPA-2 dose-dependently inhibits serotonin-induced secretion from *R. prolixus* Malpighian tubules but not secretion induced by Rhopr-CRF/DH (67). It also stimulates an elevation in cGMP content in serotonin-stimulated Malpighian tubules, but not in unstimulated Malpighian tubules. Serotonin alone decreases cGMP content of Malpighian tubules and Rhopr-CAPA-2 returns these levels to those of resting tubules (45). Furthermore, Rhopr-CAPA-2 inhibits serotonin-stimulated absorption across the anterior midgut, but in a cGMP and Ca^2+^-independent manner (8). It seems evident that Rhopr-CAPA-2 is an antidiuretic hormone in *R. prolixus*, acting on anterior midgut and Malpighian tubules to rapidly inhibit the post-prandial diuresis.

As discussed, serotonin and Rhopr-CRF/DH act synergistically on *R. prolixus* Malpighian tubules to stimulate diuresis. Rhopr-CAPA-2 inhibits serotonin-stimulated secretion but not Rhopr-CRF/DH-stimulated secretion. It does, however, reduce natriuresis elicited by either of the diuretic hormones (44). Of some importance, Rhopr-CAPA-2 eliminates the synergism produced by these two diuretic hormones. These effects may be particularly important since following termination of the rapid post-prandial diuresis, low secretion rates may be needed. Thus, once the bulk of the Na^+^-rich plasma component of the blood meal has been ejected, the insect will need to eliminate a more K^+^-rich urine following digestion of the nutrient component, including the red blood cells (44), and so Rhopr-CRF/DH may be involved in this later, slower, diuresis.

The rhodopsin-like family A GPCR for CAPA has been characterized in *D. melanogaster* (CG14575), *A. gambiae*, *R. prolixus*, *B. mori*, and predicted in several other species (68). The GPCRs are evolutionarily related to vertebrate neuromedin receptors.

The Rhopr-CAPA-R gene encodes two mRNA variants, CAPAR-1 (GU734127) and CAPAR-2 (GU734128). Sequence analysis of CAPAR-1 receptor reveals features characteristic of the rhodopsin-like family A GPCRs. Transcript expression is predominantly in the distal Malpighian tubules (60-fold higher levels than in the proximal Malpighian tubules) compared with the anterior midgut, but expression also appears in the foregut, posterior midgut, and hindgut, with the latter demonstrating lowest levels. Other tissues, such as the CNS, salivary glands, reproductive tissues, trachea, fat body, dorsal vessel, or abdominal nerves, do not exhibit any detectable levels of the receptor transcript (69).

#### Dual Diuretic/Antidiuretic Hormones

##### CCHamide2 as a Modulator of Diuresis

An intriguing recent discovery concerns the peptide CCHamide (named for its two conserved cysteines and C-terminal amidated histidine), whose receptors were previously identified in Malpighian tubules of *D. melanogaster* (26). CCHamide occurs as two paralogs, CCHa1 and CCHa2, both of which are present in the *R. prolixus* genome (14). In addition, the *R. prolixus* genome also encodes two family A GPCRs for CCHa. CCHa1 and 2 are associated with feeding behavior in *D. melanogaster* (although no studies looked at Malpighian tubule physiology), and recently Capriotti et al. (23, 32) examined the effects of CCHa2 on diuresis in *R. prolixus*. These studies revealed that the CCHa2 transcript was expressed in the CNS, but also in the anterior midgut and Malpighian tubules. Furthermore, the transcripts for the two receptors (RPRC007766 and RPRC000608) were found in the CNS and posterior midgut, with RPRC000608 also appearing in the Malpighian tubules. While CCHa2 has no effect on its own on either anterior midgut absorption or Malpighian tubule secretion, CCHa2 does have a dual effect on diuresis, blocking the effect of serotonin on absorption across the anterior midgut whilst enhancing the effect of serotonin on secretion from Malpighian tubules. This is the first example in an insect of a hormone having opposite actions (inhibition and stimulation) on different tissues involved in salt and water balance. The mode of action of CCHa2 on anterior midgut appears to be that of inhibiting the serotonin-stimulated transcellular Na^+^ transport across the anterior midgut. The authors suggest that CCHa2 modulates post-prandial diuresis and fine tunes the system, which would be necessary to respond to differences in Na^+^ content of blood meals from different host species. The effects of CCHa2 on Rhopr-CRF/DH stimulated secretion or on the synergism between serotonin and Rhopr-CRF/DH have not been examined.

### New Insights From Transcriptome Analyses

Transcriptomes and microarrays have provided molecular insights into the functional contributions of the Malpighian tubules, confirming the general model of these ion and water transport mechanisms (4, 5, 9, 10, 70). The power of these studies also lies in the unexpected, the unknown. Thus, the number and diversity of these diuretic and antidiuretic hormones and their GPCRs is ever increasing, spurred on by genetic/molecular tools that supports previous research on identified diuretic factors, along with pointing to some novel ones [see (2, 4, 5, 70)]. For example, insect tachykinins have not been tested on Malpighian tubule function in mosquitoes, but the tachykinin GPCR appears to be the most abundant of at least 17 GPCRs identified through a Malpighian tubule transcriptome of *Aedes albopictus* (4). Similarly, along with receptors for known diuretic hormones in *D. melanogaster*, are receptor transcripts for neuropeptide F, CCHamide, acetylcholine, and gamma-aminobutyric acid (GABA), along with genes associated with termination of cell signals [see (5, 26)] for which there is little understanding of their biological significance (but see above for CCHamide in *R. prolixus*). And in *Ae. albopictus*, for example, the most abundant transcript for an ion transporter is a K^+^-dependent Na^+^/Ca^2+^ exchanger (3 times more abundant than the V-type H^+^-ATPase which is considered the driver of diuresis). There is also a second K^+^-dependent Na^+^/Ca^2+^ exchanger and two Na^+^/Ca^2+^ exchangers, suggesting Ca^2+^ as a second messenger (4).

To gain further insight into the functioning of *R. prolixus* Malpighian tubules, we performed a transcriptome analysis.

## Materials and Methods

### Insects

*Rhodnius prolixus* were obtained from a long-established colony at the University of Toronto Mississauga. Insects were reared in incubators at 25°C under high humidity (~50%). The insects are fed through a latex-based feeding membrane on sterile defibrinated rabbit blood (Cedarlane Laboratories Inc., Burlington, ON, Canada) once in each instar. Non-lubricated condoms (Trojan TM, ENZ, Church and Dwight co., NJ, USA) are used as the feeding membranes. Briefly, after washing and rinsing, condoms are cut open longitudinally and stretched over the top of a 20 ml circular dish and tied in place. Afterward, the dish is filled with 20 ml of defibrinated rabbit blood using a sterile syringe connected *via* a needle through the side of the dish. Once filled, the opening is sealed, and the dish is placed on a heating plate set at 37°C for 20 minutes. The feeding jar containing the *R. prolixus* is inverted onto the membrane of the dish for insects to feed through the metal mesh lid. Fifth instars *R. prolixus* [15 days post-ecdysis (PE)] with similar feeding and body weight history were used. A group of insects was separated and allowed to take a blood meal for 20 minutes by which time the insects typically gorge at least nine times their own initial body weight. Malpighian tubules were sampled from insects at 15 d PE (unfed condition) and 3 h and 24 h post blood meal (fed condition).

### Illumina Sequencing

The Malpighian tubule RNA samples were obtained from unfed fifth instar *R. prolixus* and from insects at 3 and 24 h post blood meal. Tissues were dissected in cold, previously autoclaved, phosphate buffered saline (PBS, 6.6 mM, Na_2_HPO_4_/KH_2_PO_4_, 150 mM NaCl, pH 7.4). Three independent experiments were performed (n = 3) for each condition with each n composed of a pool of Malpighian tubules from 10 insects. RNA extraction was performed with TRIzol reagent (Invitrogen by Thermo Fisher Scientific, MA, USA), followed by DNase treatment (Millipore-Sigma, WI, USA) and then re-purified with PureLink RNA Mini Kit (Ambion by Thermo Fisher Scientific, MA, USA), as previously described (71, 72). A total amount of 1 µg RNA per sample was used as input material for the RNA sample preparations. Libraries for sequencing were generated using NEBNext Ultra RNA Library Prep Kit for Illumina (New England Biolabs, MA, USA) following the manufacturer’s recommendations. The libraries were sequenced on Illumina platforms (Illumina NovaSeq 6000) at the Novogene sequencing facility (California, USA). All the downstream analyses were performed as previously described (71, 72). RNA-seq metrics from *R. prolixus* transcriptomes for Malpighian tubules under the different feeding conditions are summarized in the first tab of **Supplementary Worksheets 1**, **2**. The data quality control showed indices expected to advance towards a high-quality transcriptome analysis. The data obtained were analyzed using gene annotation from the RproC1.3 gene set (14), and *R. prolixus* alternative annotation gene set (73). Briefly, clean reads were aligned to the reference genome using HISAT2 software, and then, HTSeq v0.6.1 was utilized to count the number of reads mapped to each gene. FPKM (expected number of Fragments Per Kilobase of transcript sequence per Millions base pairs sequenced) of each gene were calculated based on the length of the gene and number of reads mapped to the gene. The raw sequence dataset of this project is registered with the National Center for Biotechnology Information (NCBI) under PRJNA729781 BioProjects. A detailed description of our bioinformatic pipeline can be found in Leyria et al. (71). Briefly, differential expression analysis of unfed, 3 h post blood meal and 24h post blood meal was performed using the DESeq R package (1.18.0) as reported (71, 72). The resulting P values were adjusted using the Benjamini and Hochberg’s approach for controlling the False Discovery Rate (FDR). Genes with an adjusted P value < 0.05 found by DESeq2 were assigned as differentially expressed. We performed heatmap analysis to compare mRNA expression levels under the different feeding states presented by means of a color scale, showing the readcount values obtained by gene expression analysis after normalization. All the numeric information of the heatmap charts is shown in several worksheet tabs of **Supplementary Worksheet 1** (unfed *vs.* 3 h PBM) and **Supplementary Worksheet 2** (unfed *vs.* 24 h PBM).

### Validation of RNA-Seq Data

To validate differentially expressed genes obtained by Illumina sequencing, fifteen receptors were chosen, and their transcript expressions analyzed by reverse transcription quantitative real-time polymerase chain reaction (RT-qPCR) on Malpighian tubules from unfed and fed insects. Briefly, total RNA was extracted as described above and the concentration and A260/280 ratio of purified RNA measured using a spectrophotometer DS-11+ (DeNovix Inc., Wilmington, DE, USA). RNA integrity was evaluated by electrophoresis in a 1% agarose gel (FroggaBio Inc., Concord, ON, Canada). cDNAs were synthesized from 1 µg of total RNA by reverse transcription reaction using random primers and 50 U of MultiScribe™ MuLV reverse transcriptase (High-Capacity cDNA Reverse Transcription Kit, Applied-Biosystems, by Fisher Scientific, ON, Canada). The conditions of the thermal cycler were: 10 minutes at 25°C, 120 minutes at 37°C, and 5 minutes at 85°C. The cDNAs obtained were diluted 10-fold for the experiments. qPCRs were performed using an advanced master mix with super green low ROX reagent (Wisent Bioproducts Inc, QC, Canada), according to the manufacturer’s recommendations, using 4 pmol of sense and antisense primers in a final volume of 10 μl. The qPCR temperature-cycling profile was: initial denaturation 3 minutes at 95°C, followed by 39 cycles of 30 s at 94°C, 30 s at 58–60°C (depending on the pair of primers used), and 1 minute at 72°C, followed by a final extension at 72°C for 10 minutes. Each reaction contained 3 technical replicates as well as a no template and a no reverse transcriptase control. qPCR was performed using a CFX384 Touch Real-Time PCR Detection System (BioRad Laboratories Ltd., Mississauga, ON, Canada). The sequences of the primers used for amplification are shown in **Supplementary Table 1**. The primer efficiencies lay between 90-110%. For each pair of primers, a dissociation curve with a single peak was seen, indicating a single cDNA product was amplified. Quantitative validation was analyzed by the 2^−ΔCt^ method (74), using the geometric average expression of two reference genes, namely rp49 and β-actin, which were previously validated for transcript expression in Malpighian tubules of fifth instar *R. prolixus* at different nutritional conditions (30).

## Results and Discussion

### Transcriptome Analysis of *R. prolixus* Malpighian Tubules

Here, we have performed an in-depth exploration analyzing transcriptomes of the complete set of RNA transcripts that are produced by Malpighian tubules and examined changes related to feeding. It should be emphasized that the transcriptome was generated from whole Malpighian tubules and so transcripts associated with the distal and proximal portions are present. This research confirms previous findings and generates new insights about the presence/absence and changes in transcript expression in Malpighian tubules of fifth instar *R. prolixus* at key time points of the diuretic process (3 h and 24 h post blood feeding).

#### Receptors

**Figure 5:**
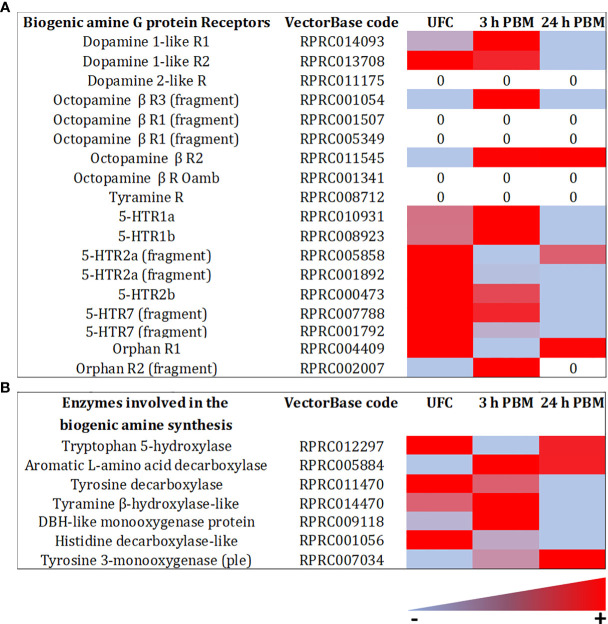
Heat map comparing the mRNA expression levels of **(A)** biogenic amine G protein- coupled receptors family A, and **(B)** the enzymes involved in biogenic amine synthesis in Malpighian tubules from insects unfed (UFC), 3 h and 24 h post blood meal (PBM). The input data is the readcount value from gene expression level analysis after normalization and is presented using a color scale, in which light blue/red represent lowest/highest expression. DESeq was used to perform the analysis. Details of transcript expression are shown in **Supplementary Worksheets 1**, **2**.

**Figure 6:**
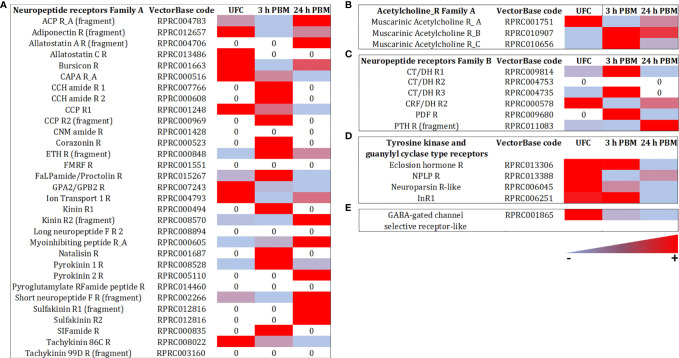
Heat map comparing the mRNA expression levels of **(A)** Neuropeptide receptors family A, **(B)** Acetylcholine receptors family A, **(C)** Neuropeptide receptors family B, **(D)** Tyrosine kinase and guanylyl cyclase type receptors, and **(E)** GABA-gated channel selective receptor-like in Malpighian tubule from insects unfed (UFC), 3 h and 24 h post blood meal (PBM). The input data is the readcount value from gene expression level analysis after normalization and is presented using a color scale, in which light blue/red represent lowest/highest expression. DESeq was used to perform the analysis. Details of transcript expression are shown in **Supplementary Worksheets 1**, **2**.

**Figure 7:**
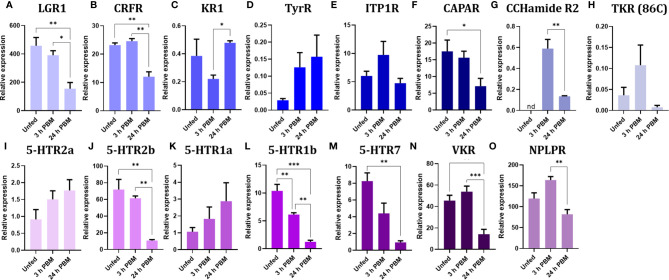
Verification of transcriptome analysis by RT-qPCR for G protein-coupled receptors (GPCRs) and receptor tyrosine kinases (RTK) in Malpighian tubules of unfed and fed insects. Fifth instar insects were dissected at 15 days post-ecdysis as representative days of the unfed condition (unfed). One group of insects was allowed to gorge, and mRNA expression assayed at 3 and 24 h post blood meal (h PBM). The expression of transcripts to thirteen GPCRs **(A–M)** and two RTKs **(N, O)** was quantified using RT-qPCR and the ΔCt method. The y axis represents the relative expression by 1000 copies of reference genes, obtained *via* geometric averaging using Rp49 and β-actin. The results are shown as mean ± SEM (n=4-5). Statistical analysis was performed using a one-way ANOVA and Tukey’s test for post-hoc analysis; p-values are listed on the graph (****p < 0.0001; ***p < 0.001; **p < 0.01; *p < 0.05; nd, not detected). Graphs and statistical tests were performed using GraphPad Prism 7. LGR1, GPA2/GPB2 receptor; CRFR, corticotropin-releasing factor-related diuretic hormone receptor 2; KR1, kinin receptor 1; TyrR, tyramine receptor; ITP1R, ion transport peptide 1 receptor; CAPAR, CAPA receptor 1 isoform A; CCHamideR2, CCH amide2 receptor; TKR (86C), Tachykinin 86C receptor; 5-HTR2a, 5-HT receptor 2a; 5-HTR2b, 5-HT receptor 2b; 5-HTR1a, 5-HT receptor 1a; 5-HTR1b, 5-HT receptor 1b; 5-HTR7, 5-HT receptor 7; VKR, neuroparsin receptor-like (Venus kinase receptor); NPLPR, neuropeptide-like precursor 1 receptor.

Hormonal signaling is amplified *via* second messengers, and lower receptor expression may be expected relative to transcripts involved in other cellular activities. Therefore, since the neuroendocrine control of the insect excretory system is mainly transduced *via* GPCRs, the expression of these receptors is relatively low when compared, for example, to other kinds of receptors or epithelial transporters (**Supplementary Worksheets 1**, **2**). The GPCR transcripts encoding the *R. prolixus* receptors Rhopr5HTR2b, Rhopr-CRF/DH-R2B, and Rhopr-CAPA-R1, which have previously been shown to be expressed in the Malpighian tubules by qPCR, are all highly abundant in the transcriptome (**Figures 5**, **6** and **Supplementary Worksheets 1**, **2**). Of these, Rhopr5HTR2b is the most highly expressed receptor, and interestingly, of the five serotonin receptor sub-types present, is also 100-400 fold more abundant. Furthermore, the transcript for Rhopr-CT/DH-R2B is of very low abundance, and that of Rhopr-CCHa2R even lower and not evident in Malpighian tubules from an unfed insect in the transcriptome or by qPCR (**Figure 7**). Interestingly, none of the receptors for serotonin, Rhopr-CRF/DH or Rhopr-CAPA are within the most abundant receptors expressed in the transcriptome.

Of note is the discovery of GPCRs for ligands not previously tested for biological activity at the Malpighian tubules of *R. prolixus* (**Figure 6**; **Supplementary Worksheets 1**, **2**). Indeed, the most abundant receptor transcript is that of the leucine-rich repeat-containing G protein-coupled receptor 1 (LGR1) for the glycoprotein hormone, GPA2/GPB5, which is approximately 6-fold more abundant than the Rhopr5HTR2b and 60-fold more abundant than Rhopr-CRF/DH-R2B. Another receptor transcript that is highly abundant is the receptor-type guanylate cyclase for the neuropeptide-like precursor peptide (NPLPR). LGR1 and NPLPR transcripts are also confirmed by qPCR (**Figure 7**). Other previously unappreciated receptors that might be involved in Malpighian tubule function include orthologs of a neuroparsin receptor-like (Venus kinase receptor (VKR)), parathyroid hormone-like receptor, adiponectin receptor, short neuropeptide F receptor (sNPFR), dopamine 1-like R2, ion transport peptide receptor (ITPR), GABA receptor, and the insulin receptor 1 (IR1). Low levels for the muscarinic acetylcholine receptor and Rhopr-KR are also evident, as well as Rhopr-CT/DH-R1, previously reported to be missing from Malpighian tubules (39).

#### Endogenous Hormones

A novel discovery was made in *D. melanogaster* by Blumenthal (21) whereby tyramine, synthesized from tyrosine in the principal cells of the Malpighian tubules (*via* tyrosine decarboxylase), is released and acts *via* a tyramine GPCR (CG7431) located on the basolateral membrane of the neighboring stellate cells, to regulate Cl^-^ conductance. Tyramine acts, therefore, in an autocrine or paracrine way. Adding to the possibility of autocrine/paracrine signaling in Malpighian tubules is the observation from transcriptome analysis that neuropeptide precursor transcripts are also expressed in Malpighian tubules of larval *Trichoplusia ni* (2).

**Figure 8:**
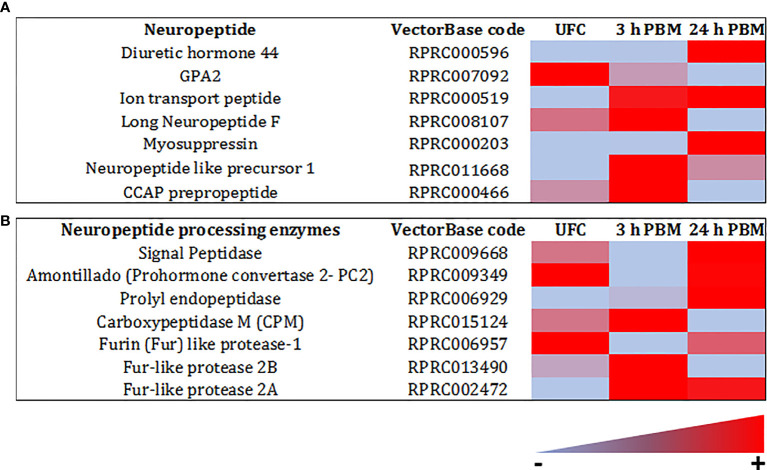
Heat map comparing the mRNA expression levels of **(A)** neuropeptides, and **(B)** neuropeptide processing enzymes in Malpighian tubules from insects unfed (UFC), 3 h and 24 h post blood meal (PBM). The input data is the readcount value from gene expression level analysis after normalization and is presented by means of a color scale, in which light blue/red represent lowest/highest expression. DESeq was used to perform the analysis. Details of transcript expression are shown in **Supplementary Worksheets 1**, **2**.

Similarly, in *R. prolixus* tubules, we identify high abundance for transcripts for neuropeptide processing enzymes and biogenic amine processing enzymes, and neuropeptide precursors (**Figures 5**, **8** and **Supplementary Worksheets 1**, **2**). In terms of neuropeptide precursors, there is an abundance of the GPA2 transcript, and lower abundance of transcripts for Rhopr-CRF/DH, ITP, Long NPF, myosuppressin, and NPLP1(**Figure 8** and **Supplementary Worksheets 1**, **2**). The transcript for tachykinin has also previously been shown to be present (75). In terms of biogenic amines and other transmitters, by far the most abundant transcript for a processing enzyme is that of histidine decarboxylase which converts histidine to histamine. This transcript is several thousand-fold greater than other less abundant enzyme transcripts, although enzymes in the dopamine, nor-epinephrine, and octopamine pathways are evident (**Figure 5** and **Supplementary Worksheets 1**, **2**). Because of the broad repertoire of these transcripts expressed by the Malpighian tubules and involved in synthesis and processing of biogenic amines, peptides, and other transmitters, it is clear that a specific and controlled local system could also be modulating diuresis.

#### Second Messengers

**Figure 9:**
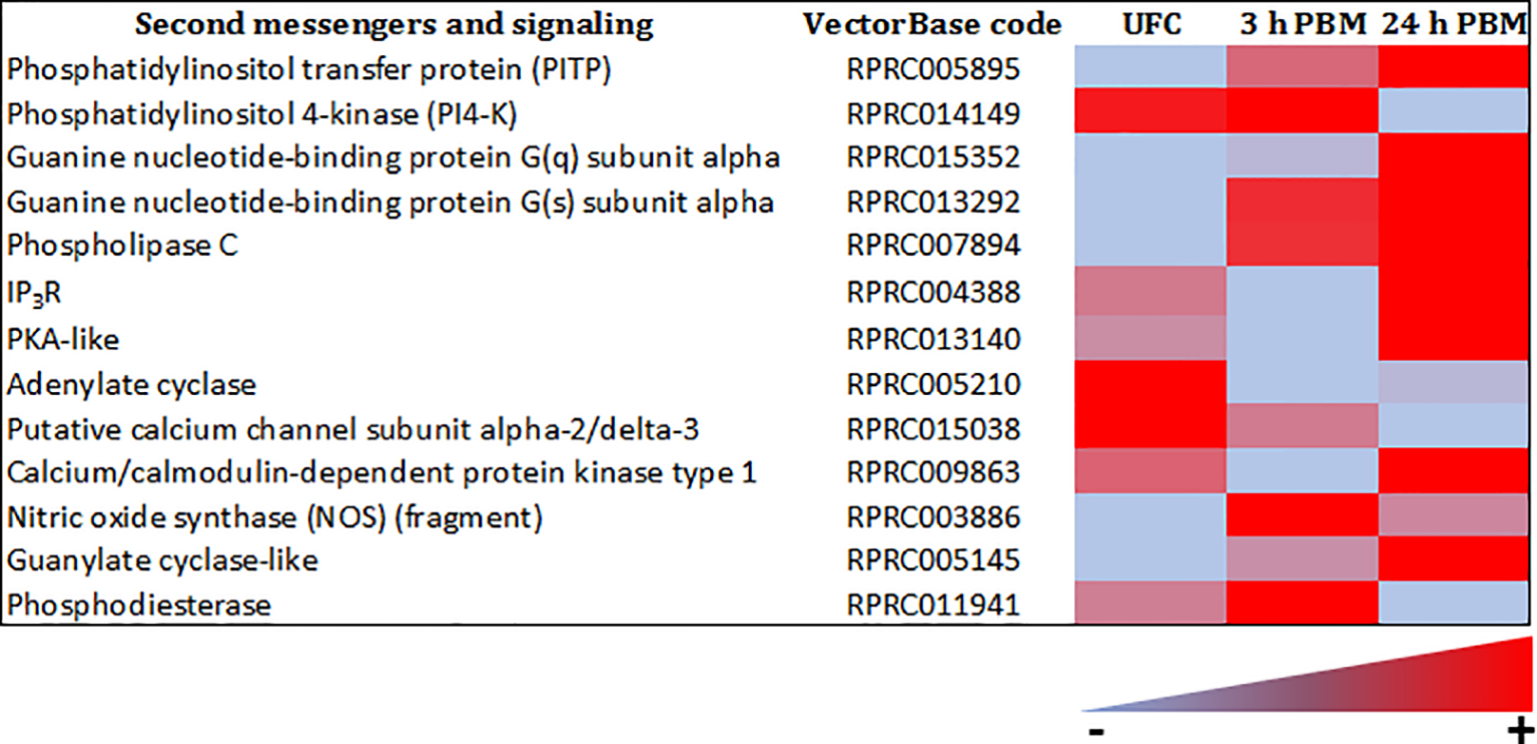
Heat map comparing the mRNA expression levels of second messengers and signaling molecules in Malpighian tubules from insects unfed (UFC), 3 h and 24 h post blood meal (PBM). The input data is the readcount value from gene expression level analysis after normalization and is presented by means of a color scale, in which light blue/red represent lowest/highest expression. DESeq was used to perform the analysis. Details of transcript expression are shown in **Supplementary Worksheets 1**, **2**.

The sensitivity of a cell to a specific hormonal response is determined not only by the expression of its receptor, but also by the level of metabolic activity that the cell exhibits, which is ultimately influenced by signaling systems mediated by second messengers. The Malpighian tubule transcriptome of *R. prolixus* reveals abundant transcript expression for a variety of second messengers, including adenylate cyclase, PKA-like, IP_3_ receptor (IP_3_R), guanine nucleotide-binding proteins, PLC, phosphodiesterase (PDE), phosphatidylinositol pathway, guanylate cyclase-like, and nitric oxide synthase (NOS) (**Figure 9** and **Supplementary Worksheets 1**, **2**). Clearly, the machinery is present in Malpighian tubules for the second messengers that are activated *via* the currently known diuretic and antidiuretic hormones.

Although obviously not considered as second messengers, it is worth noting that innexin proteins (invertebrate connexins) form gap junction channels participating in cellular communication. The Malpighian tubule transcriptome analysis (**Supplementary Worksheets 1**, **2**) shows the presence of transcripts for innexins, which could potentially contribute to Ca^2+^ wave propagation. Innexin gap junctions propagate Ca^2+^ waves in the *C. elegans* intestine (76). In addition, the role of innexins in coordinating and regulating the rapid diuretic effects of neuropeptides was already suggested in *A. aegypti* (77).

#### Epithelial Transporters

**Figure 10:**
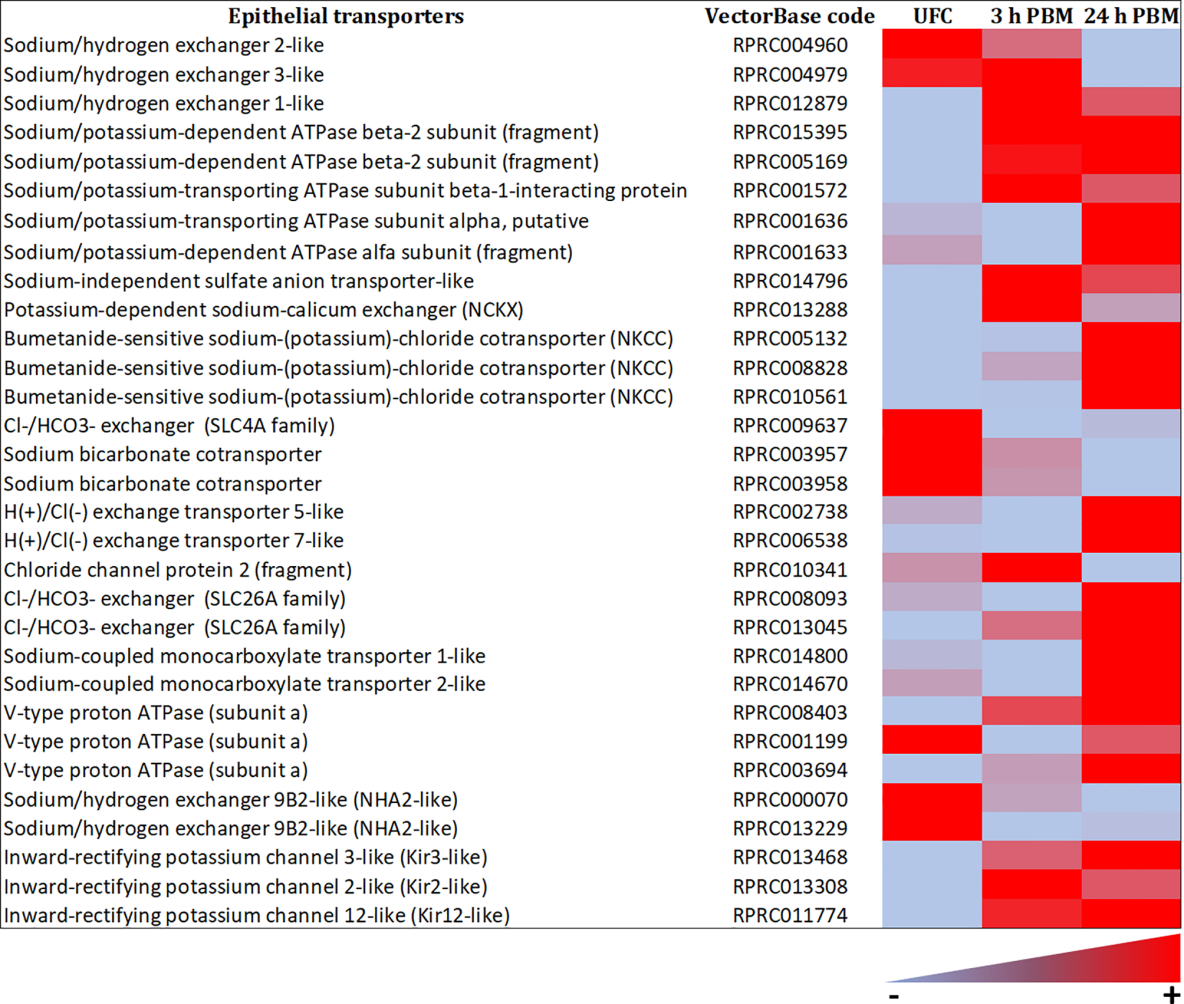
Heat map comparing the mRNA expression levels of epithelial transporters in Malpighian tubules from insects unfed (UFC), 3 h and 24 h post blood meal (PBM). The input data is the readcount value from gene expression level analysis after normalization and is presented by means of a color scale, in which light blue/red represent lowest/highest expression. DESeq was used to perform the analysis. Details of transcript expression are shown in **Supplementary Worksheets 1**, **2**.

**Figure 11:**
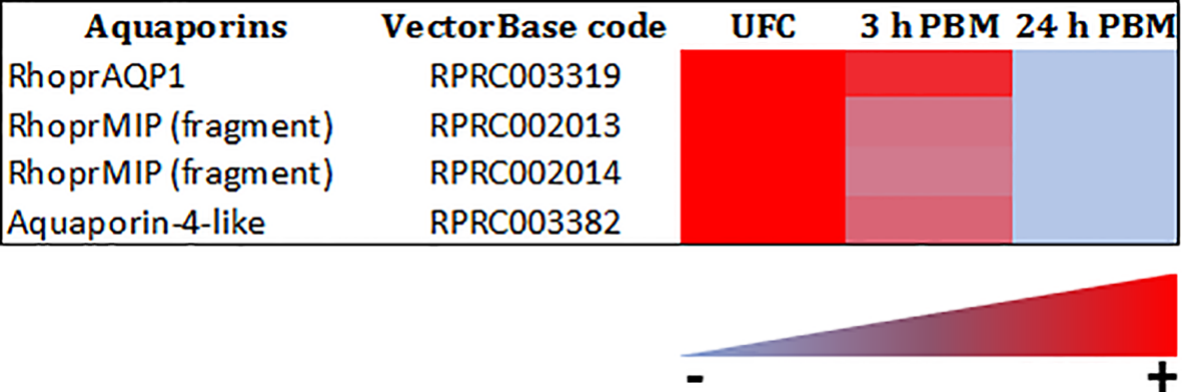
Heat map comparing the mRNA expression levels of aquaporins in Malpighian tubules from insects unfed (UFC), 3 h and 24 h post blood meal (PBM). The input data is the readcount value from gene expression level analysis after normalization and is presented by means of a color scale, in which light blue/red represent lowest/highest expression. DESeq was used to perform the analysis. Details of transcript expression are shown in **Supplementary Worksheets 1**, **2**.

The *R. prolixus* Malpighian tubule transcriptome reveals numerous transcripts that encode for ion and water transport mechanisms (**Figures 10**, **11** and **Supplementary Worksheets 1**, **2**), including V-type H^+^-ATPase subunits, cation-proton antiporters, cation Cl^-^ cotransporters such as NKCC, HCO_3_ exchangers, barium-sensitive K^+^ channels, aquaporins, and Major Intrinsic Proteins (MIPs). These are in very high abundance (considerably higher than those for the receptors) and are needed to generate the very high rate of active fluid transport required in a short time. The transporters evident in the transcriptome match those in the model for fluid secretion by Malpighian tubules of *R. prolixus* (33, 34), and to models for other insect Malpighian tubules (33). The transporters involved in fluid secretion are quite conserved across insect species. Interestingly though, the transcriptome data suggest a lack of vertebrate-like H^+^/K^+^-ATPases in *R. prolixus*, which were suggested to be involved in K^+^ reabsorption by the proximal tubules (78). Thus, this H^+^/K^+^-ATPase in *R. prolixus* tubules could be a pump with lower sequence conservation to H^+^/K^+^-ATPase of the gastric mucosa of vertebrates, or replaced by other transport mechanisms, such as an NHA linked to a V-type H^+^-ATPase.

The transcript for an NCKX is also present in the *R. prolixus* Malpighian tubule transcriptome, although not as highly expressed as that of *Ae. albopictus.* The NCKX plays critical roles in regulating intracellular Ca^2+^ levels and since, as described earlier, intracellular Ca^2+^ waves induced by serotonin drive fluid transport in *R. prolixus*, may present a mechanism for homeostatic control of cytosolic Ca^2+^ levels. In addition, there are transcripts for Ca^2+^ and Cl^-^ channels and $\text{N}{\text{a}}^{+}{\text{/HCO}}_{3}^{-}$ cotransporters (**Figure 10** and **Supplementary Worksheets 1**, **2**). Moreover, considering synergism between serotonin and Rhopr-CRF/DH on Malpighian tubule secretion, amiloride inhibits secretion induced by serotonin, but not secretion induced by RhoprCRF/DH (45). The cation/proton antiporter (CPA) family is ubiquitous and best known for Na^+^/H^+^ exchangers (NHEs) and, more recently discovered, the Na^+^/H^+^ antiporters (NHAs). In mosquitoes, three NHE isoforms are known to be present: NHE1, NHE2 and NHE3 (79, 80). NHE2 is resistant to amiloride, while NHE3 and NHE1 are amiloride-sensitive. Interestingly, both types of NHEs are found in the *R. prolixus* fifth instar Malpighian tubule transcriptome, suggesting that synergism between serotonin and Rhopr-CRF/DH on Malpighian tubule secretion could also in part be achieved by activating two different types of NHEs (**Figure 10** and **Supplementary Worksheets 1**, **2**). In addition, *D. melanogaster* and *Anopheles gambiae* each have two NHA genes, Nha1 and Nha2 [see (80, 81)]. The NHAs are hypothesized to act as the partner exchangers that colocalize with V-ATPase and use the proton electrochemical gradient to achieve transepithelial transport of Na^+^ and K^+^ (82). In *R. prolixus*, NHA2-like transcripts are found in the Malpighian tubule transcriptome, in unfed and fed insects. Since it is possible that there is no vertebrate-like H^+^/K^+^-ATPase in *R. prolixus*, NHA2-like might collaborate with the V-type H^+^ ATPase to mediate transepithelial active transport of Na^+^ and K^+^ (**Figure 10** and **Supplementary Worksheets 1**, **2**).

#### Changes After a Blood Meal

Analyses on the abundance of transcripts does not in of itself tell a complete story. Insight must also be obtained by examining how transcript expression changes with blood feeding; that is, which transcripts are unaltered, which transcripts are upregulated, and which transcripts are downregulated. However, as the control of the neuroendocrine system involves diuretic and antidiuretic hormones acting in concert and individually over a short period of time, the number of differentially expressed genes is low with respect to all those reported here.

After *R. prolixus* takes a blood meal, there is a trend of significantly down regulating the most abundant transcripts for receptors at 24 h post blood meal, including LGR1, CRF/DH-R, CAPA-R, 5HTR-2b, 5HTR-1b, 5HTR-7, VKR, and NPLPR. This down regulation was observed in the transcriptome and confirmed by qPCR (**Figures 5**–**7** and **Supplementary Worksheets 1**, **2**). The GABA-gated channel receptor is the most highly expressed receptor in unfed *R. prolixus* and is significantly down regulated at 3 h and 24 h. One exception includes the sNPFR which is significantly up regulated at 24 h. It is worth repeating that there is no known function for LGR1, VKR, NPLPR, sNPFR, or GABA-R or their ligands in the control of *R. prolixus* Malpighian tubules. However, GPA2/GPB5 and neuroparsin signaling, mediated by their putative receptors, LGR1 and VKR, respectively, have been suggested to have potential roles as antidiuretic hormones in other insects, acting on the hindgut (83–85). The fact that both receptors are more highly expressed in Malpighian tubules of unfed *R. prolixus* relative to fed insects might indicate a potential role of this signaling pathway in water conservation in unfed *R. prolixus*. Also, we detected the expression of Tachykinin 86C receptor (TKR) in Malpighian tubules and although the levels are low, they are higher in unfed insects. Interestingly, in *D. melanogaster* DTKR triggers insulin signaling in Malpighian tubules, influencing the insect’s responses to desiccation and oxidative stress (22). Here, we also identified IR1 transcript expression in the Malpighian tubules. In addition, the GABA-R has also been identified in *D. melanogaster* Malpighian tubules (26).

Receptor transcripts that are in very low abundance relative to the above, tend to show no significant change between unfed, 3 h or 24 h post blood meal (**Figures 5**–**7** and **Supplementary Worksheets 1**, **2**). Exceptions include the transcripts for KR1 which are significantly down regulated at 3 h, but not at 24 h; and CCHaR2, a transcript which is not evident in the transcriptome of unfed or 24 h fed insects, or by qPCR of Malpighian tubules from unfed insects, but can be seen by qPCR in very small amounts in 3 h fed, and even smaller amounts in 24 h fed. This low abundance does seem sufficient to enable CCHa2 to enhance serotonin-induced secretion by Malpighian tubules (23). Parathyroid hormone receptor (PTHR) is upregulated at 24 h in the transcriptome. In mammals, parathyroid hormone signaling regulates the serum calcium concentration through its effects on kidney and intestine, among others. Interestingly, along with Ca^2+^ signaling being involved in diuresis, Ca^2+^ from the blood meal is sequestered into Malpighian tubule cells as concretion bodies, from where they can be released back into the hemolymph for Ca^2+^ homeostasis (86, 87). Perhaps PTHR is involved in this.

In terms of possible endogenous hormones, an intriguing observation is the very large abundance of transcript for histidine decarboxylase, which is significantly down regulated at 24 h post blood meal (**Figure 5** and **Supplementary Worksheets 1**, **2**). This enzyme is the only enzyme used for the synthesis of histamine. Histamine is the neurotransmitter of photoreceptors in insects and other arthropods. As a photoreceptor transmitter, histamine acts on ligand-gated chloride channels, and elsewhere in the CNS there are receptors similar to the mammalian H1 GPCRs. There do not appear to be any reports of histamine in any other tissues in insects. Its possible presence in Malpighian tubules could suggest an involvement in diuresis, and indeed an early study has shown histamine to induce a small increase in secretion in locust Malpighian tubules (88). Of course, Malpighian tubules participate in the immune response of insects, and so perhaps histamine might have some involvement in this aspect in *R. prolixus*. In addition, the transcript for GPA2 is downregulated at 3 h and 24 h post blood meal, with the transcript for Rhopr-CRF/DH upregulated at 24 h, that of long NPF downregulated at 24 h, and that of mysuppressin upregulated at 24 h (**Figure 8**). Again, there is currently no known function for GPA2, LNPF or myosuppressin in *R. prolixus* Malpighian tubule physiology. In addition, transcripts for neuropeptide processing enzymes are altered by blood feeding, with the upregulation of signal peptide and propyl endopeptidase transcripts at 24 h, upregulation of Furin-like protease- 2A at 3 h and 24 h, but downregulation of Furin-like protease-1 at 3 h and 24 h, indicating that, at least up to 24 h post blood meal there could be a constant local synthesis of the peptides involved in diuretic and antidiuretic actions (**Figure 8**).

The transcripts for second messengers show a variable pattern of differentiated expression following a blood meal (**Figure 9** and **Supplementary Worksheets 1**, **2**). The diuretic hormones initially act *via* cAMP and the transcripts for adenylate cyclase are downregulated at 3 h and 24 h post blood meal, when the rapid post-prandial diuresis is reduced. The antidiuretic hormone acts *via* cGMP and the transcripts for guanylate cyclase and NOS are upregulated at 3 h and 24 h, and the PDE transcript has a slight upregulation at 3 h. Guanine nucleotide-binding protein G(s) subunit and phosphatidylinositol transfer protein are upregulated at 3 h and 24 h, guanine nucleotide-binding protein G(q) subunit up at 24 h and phosphatidylinositol 4-kinase down at 24 h. The PLC subtypes also vary and PKA is down regulated at 3 h. Of some significance also for the termination of diuresis, is the down regulation of aquaporin transcripts, which at 24 h indicates a 10-fold decrease in Rhopr-AQP 1transcript and an approximately 6-fold decrease in Rhopr-MIP transcript (**Figure 11** and **Supplementary Worksheets 1**, **2**). Interestingly, in contrast to *Ae. albopictus* (4) the innexin transcripts in *R. prolixus* are upregulated at 3 h and 24 h post blood meal, indicating that the coupling between epithelial cells outlasts that which may be needed during rapid post-prandial diuresis (**Supplementary Worksheets 1**, **2**).

The decrease in transcript expression of AQPs after 24 h post feeding suggests a decreased capacity for secretion of water. In terms of transporters, it is also interestingly to note that the most abundant V-type H^+^ ATPase subunit a, belonging to the V0 integral protein complex and which drives fluid transport in the distal Malpighian tubules, is downregulated at 3 h and 24 h post blood meal when diuresis is greatly reduced, while others, including V-type H^+^ ATPase subunits H, D, B, and G from the peripheral protein complex V1, show variable changes. SLC4A, NHE2 (resistant to amiloride), NHE3 (amiloride sensitive) and NHA2 exchangers, as well as calcium channel subunits, are also regulated in a similar fashion to AQPs, indicating additional potential mechanisms for reducing ion transport.

The Na^+^/K^+^-ATPase (subunit beta) is upregulated at 3 h and 24 h post blood meal as are various cotransporters (**Figure 10** and **Supplementary Worksheets 1**, **2**), including NKCC at 24 h. SLC 4A and 26A transport families for $\text{C}{\text{l}}^{-}{\text{/HCO}}_{3}^{-}$ transport are upregulated at 3 h and 24 h post blood meal. These transporters are important in the proximal Malpighian tubules for reabsorption of KCl and their upregulation by feeding suggests that KCl reabsorption continues after rapid post-prandial diuresis has declined. Inward rectifying K^+^ channel transcripts are upregulated at 3 h and 24 h post blood meal, whereas Cl^-^ channel subunit expression is unchanged by feeding. H^+^/Cl^-^ exchange transporter 5, and 7-like are upregulated 24 h post blood meal, and the Ca^2+^ channel subunits are downregulated at 24 h post blood meal (**Figures 9**, **10** and **Supplementary Worksheets 1**, **2**). Transcripts for monocarboxylate transporters are evident, which typically transport organic solutes such as lactate and pyruvate. These transporters are enriched in *D. melanogaster* tubules and one of them has been suggested to be a kynurenine transporter (89). It is worth emphasizing that so far, the models have examined the rapid post-prandial diuresis in *R. prolixus*, and it is likely that other aspects of Malpighian tubule function occur over a longer time-course; for example, when the red blood cells are digested and contribute excess K^+^ which may need to be eliminated, or where waste substances and toxins may need to be eliminated.

## Final Thoughts

The present study is the first to examine the transcriptome of the Malpighian tubules of *R. prolixus*, and therefore the first to examine changes in transcript expression induced by feeding. We have concentrated on the ligands, receptors, second messengers and transporters that might control rapid post-prandial diuresis but are cognizant of the fact that Malpighian tubules are much more than transporting epithelia. And so, there are gaps in our knowledge on the involvement of diuretic/antidiuretic hormones at times other than after gorging, and gaps in our appreciation of the central importance of Malpighian tubules in stress responses, detoxification processes, and immune responses.

Until relatively recently, our understanding of diuresis in insects relied heavily on *in vitro* work, which has indeed enabled models to be developed on the neurohormonal control of diuresis. Access to the insect genomes that have been sequenced over the last 20 years or so, and advances in molecular techniques, have, however, revolutionized insect (neuro)endocrinology, and functional genomics and genetics, including gene micro-arrays, transcriptomes, mutations, double stranded RNA (dsRNA) and sophisticated peptidomics (‘omics in general) are enabling complex regulatory processes to be exquisitely dissected and defined (2, 4, 9–13). So too, the basic model of post-prandial diuresis in *R. prolixus*, with its diuretic and antidiuretic hormones, coupled to the second messengers cAMP, cGMP and IP_3_, and in turn acting *via* predicted ion channels, transporters and aquaporins is here validated and confirmed by transcriptomics; but transcriptomics has also revealed how much more still needs to be understood.

Thus, the Malpighian tubules might be a rich source of endogenous hormones that may or may not act as autocrine/paracrine factors; tubules may signal to other parts of the insect. An increasing number of receptor types suggests an increasing number of ligands, which again may control post-prandial diuresis, or may control some other functions of the Malpighian tubules such as stress tolerance, detoxification processes, and/or immunity. Most of these have not been tested for biological relevance. For example, what is the function of GABA receptors, of histamine (suggested by the abundant presence of the histidine decarboxylase enzyme transcript), of LGR1, or indeed of PTHR? Could histamine be involved in the immune response in *R. prolixus*? Also, PTHR is interesting, since the ligand (consensus sequence PXXXamide) for this receptor has only recently been identified in insects, particularly in *Tribolium castaneum* (90), with the receptor expressed in a variety of tissues including the gut (Malpighian tubules were not tested). In *T. castaneum* this signaling pathway appears to regulate cuticle formation and influence the number of eggs produced and their hatching rate. PTH is involved in bone remodeling and Ca^2+^ metabolism in vertebrates and this might be of relevance, because, as mentioned earlier, *R. prolixus* must cope with the large amounts of Ca^2+^ ingested in its blood meal. This Ca^2+^ is accumulated at high concentrations in the cells of the distal Malpighian tubules but not the proximal Malpighian tubules; very little is excreted from the body (86, 87). Ca^2+^ appears to accumulate in intracellular membrane-bound concretion bodies, which are therefore likely sites of calcium deposition. Ca^2+^ deposited in the tubules is also readily exchangeable, but the efflux preferentially passes to the hemolymph side of the tubule epithelium, and not into the lumen. Thus, Malpighian tubules would appear to participate in Ca^2+^ homeostasis. Could the PTHR be involved in maintaining hemolymph Ca^2+^ levels within a narrow physiological range, as it does in serum of vertebrates?

Finally, access to these advanced tools is accelerating our understanding of receptors such as the GPCRs and receptor tyrosine kinases, with the importance of receptors highlighted by the fact that 50% of current human drugs target GPCRs or GPCR-related genes, and this potential for drug discovery is being extended to novel pest-control strategies (91). G protein-coupled receptors constitute a very large family of proteins that include olfactory and gustatory receptors, rhodopsin-like and receptors for neurotransmitters, neuropeptides, biogenic amines, and peptide hormones. Ultimately, this large number and diversity of ligands and their receptors leads to a versatile and flexible messaging system, that enables them to function in multiple ways - as neurotransmitters, neurohormones, and neuromodulators [see (19)].

As seen, such advances have been brought to bear on the neurohormonal control of osmotic and ionic balance in insects and it is reasonable to conclude that there is much to be discovered. A comprehensive understanding of osmotic and ionic balance will require knowledge of the factors controlling these processes *in vivo* and how their release is controlled, coordinated, and monitored. Some other interesting questions include the matter of multiple diuretic hormones and why and when these diuretic hormones are co-localized? It is possible that there are multiple diuretic hormones to provide synergy and/or because they each have different physiological effects and/or are released at different times. Thus, they can act synergistically or independently.

Clearly, much is known about the neuroendocrine control of fluid secretion by the Malpighian tubules. Transcriptomics has validated and confirmed the general model but has also emphasized that there is much more to be learned and much that we do not understand. Importantly, though, transcriptomics has identified novel avenues for future research.

## Data Availability Statement

All datasets presented in this study are included in the article/**Supplementary Material**. The RNA-seq data generated for this study is available from the National Center for Biotechnology Information (NCBI) database under PRJNA729781 BioProject.

## Author Contributions

IO, AL, JL, and AA-D designed the experiments and mapped out the manuscript. JL and AA-D prepared tissues for illumina sequencing, performed in silico analysis, and RT-qPCR experiments. IO and AL wrote the initial draft of the manuscript and JL, AA-D, and AL prepared the figures. JL and AA-D reviewed and contributed to the writing of the final manuscript. All authors contributed to the article and approved the submitted version.

## Funding

This work was supported by the Natural Sciences and Engineering Research Council of Canada Discovery Grants to AL (RGPIN-2019-05775) and IO (RGPIN-2017-06402).

## Conflict of Interest

The authors declare that the research was conducted in the absence of any commercial or financial relationships that could be construed as a potential conflict of interest.

## Publisher’s Note

All claims expressed in this article are solely those of the authors and do not necessarily represent those of their affiliated organizations, or those of the publisher, the editors and the reviewers. Any product that may be evaluated in this article, or claim that may be made by its manufacturer, is not guaranteed or endorsed by the publisher.

## Abbreviations

BAPTA-AM, 1,2-bis(o-aminophenoxy)ethane-N,N,N′,N′-tetraacetic acid; cAMP, cyclic adenosine monophosphate; cGMP, cyclic guanosine monophosphate; CHO, Chinese Hamster Ovary; CNS, central nervous system; CRF/DH, corticotropin-releasing factor-related diuretic hormone; CT/DH, calcitonin-related diuretic hormone; ds RNA, double stranded RNA; DUM, dorsal unpaired median; GABA, gamma-aminobutyric acid; GPCR, G protein-coupled receptor; HEK293, Human embryonic kidney – 293; CNG, cyclic nucleotide-gated channel; IP_3_, inositol 1,4,5-trisphosphate; LGR 1, leucine rich-repeat G protein-coupled receptor; ITPR, ion transport peptide receptor; IR 1, insulin receptor 1; MTGM, mesothoracic ganglionic mass; NHAs, Na^+^/H^+^ antiporters; NHEs, Na^+^/H^+^ exchangers; NO, nitric oxide; NOS, nitric oxide synthase; NPLP, neuropeptide-like precursor peptide; OMP, Ovary Maturating Parsin; PDE, phosphodiesterase; PKA, protein kinase A; PLC, phospholipase C; PTH, parathyroid hormone; sNPFR, short neuropeptide F receptor; TKR, tachykinin receptor; VKR, Venus kinase receptor; 5HT, 5-hydroxtryptamine (serotonin); 8-Br-cAMP, 8-Bromoadenosine 3’,5’-cyclic adenosine monophosphate.
