# Supplementary material for: Fluid Secretion by Malpighian Tubules of Rhodnius prolixus: Neuroendocrine Control With New Insights From a Transcriptome Analysis
Source: Front Endocrinol (Lausanne). 2021 Aug 26;12:722487. doi: 10.3389/fendo.2021.722487 (PMC8426621; doi:10.3389/fendo.2021.722487)
Supplement: Supplementary file 3 [file Table_1.docx]

**Supplementary Table 1. Gene specific primers.**

| Accession number | Quantitative PCR (5´-3´) | |
| --- | --- | --- |
| Primers | Product size | Sequence (5’🡪3’) |
| 5-HT1A_Forward (RPRC010931) | 212 | CACACCGTCCAATGTGAGTC |
| 5-HT1A_Reverse R |  | ATGGCAACCAACAGACAACA |
| 5-HT1B_Forward (RPRC008923) | 178 | CTGCTGGTGGTAATGGAGGT |
| 5-HT1B_Reverse R |  | GTCGTGGGATCTGCTTCAAT |
| 5-HT2A_Forward (RPRC001892) | 164 | GCAAGTGTAGCCGATGGAAT |
| 5-HT2A_Reverse R |  | TGATGCTGTACCCGGTGATA |
| 5-HT2B_Forward (RPRC000473) | 237 | CTTAGACGCGACGATCACAA |
| 5-HT2B_Reverse R |  | TTGTGCTGCTTCTTCCATTG |
| 5-HT7_Forward (RPRC001792) | 181 | GTAGCACGAGTGAGCAACGA |
| 5-HT7_Reverse R |  | TTATAATGCCGAGCGTTGTG |
| VKR1_Forward (RPRC006045) | 214 | CTTCGGATGGTGGGTCTAAA |
| VKR1_Reverse |  | ACAGCAAATCGATTCCAAGG |
| CAPAR1_Forward (RPRC000516) | 107 | TGCGTGGAAACGTACATGGA |
| CAPAR1_Reverse |  | GAACGGAGCCCAGCATATGA |
| IT1R_Forward (RPRC004793) | 159 | CGAGACCCGTTATTCATCGT |
| IT1R_Reverse |  | GTCGGATATGGCCAAAGAGA |
| Kinin R1_Forward (RPRC000494) | 191 | TGCTCCACCCTCAAAATTAAGA |
| Kinin R1_Reverse |  | ACCAACATGATCATTTTCTTCAACA |
| CCHamideR2_Forward (RPRC000608) | 171 | GTATCCTGAACGCCCAGCAA |
| CCHamdeR2_Reverse |  | ATTACCAGCAGATCGCCCAA |
| CRFR2_Forward (RPRC000578) | 155 | ACTGCTCTTGGTTTGGCAGT |
| CRFR2_Reverse |  | GCCGGGTCTGTTTTAACGTA |
| Tyr1-R_Forward (RPRC008712) | 113 | GGAGAGACGAGCCGCAC |
| Tyr1-R_Reverse |  | CTGATGGACAGCAAACCGAAC |
| NPLP_Forward (RPRC008712) | 168 | CTACCACACGGGCGATTACT |
| NPLP_Reverse |  | TCCGAGCAAACGTAGGAACT |
| LGR1_Forward (RPRC007243) | 224 | CCGCCAGACACCAGAAATAC |
| LGR1_Reverse |  | TTCGTGAATCATGTGTTCTTCA |
| TKR_Forward (RPRC008022) | 212 | TCAACAACTACCTGGCAAA |
| TKR_Reverse |  | ACTGGGTTTGTGTGTAATGG |
| Actin_Forward | 289 | AGAGAAAAGATGACGCAGATAATGT |
| Actin_Reverse |  | ATATCCCTAACAATTTCACGTTCG |
| Rp49_Forward | 150 | GTGAAACTCAGGAGAAATTGGC |
| Rp49_Reverse |  | AGGACACACCATGCGCTATC |
